# Effects of painful stimulation and acupuncture on attention networks in healthy subjects

**DOI:** 10.1186/1744-9081-9-23

**Published:** 2013-06-07

**Authors:** Gang Liu, Hui-juan Ma, Pan-pan Hu, Yang-hua Tian, Shen Hu, Jin Fan, Kai Wang

**Affiliations:** 1 Department of Neurology, The First Hospital of Anhui Medical University, Hefei, Anhui Province, P. R. China; 2 Department of Psychology, Queens College, City University of New York, New York, NY 10029, USA; 3 Departments of Psychiatry and Neuroscience, Mount Sinai School of Medicine, New York, NY 10029, USA

**Keywords:** Painful stimulation, Acupuncture, Attention networks, Alerting network, Orienting network, Executive control network

## Abstract

Pain is a subjective sensory and emotional experience, and it has been reported that many different brain regions are regulated by pain, and that pain can impact attention. Acupuncture is an important treatment component of Chinese traditional medicine, and has been used for thousands of years to treat a wide variety of conditions. Although several studies have shown that acupuncture improves consciousness, the precise impact of both acupuncture and painful stimulation on attention is unclear. Are all of the attention networks modulated, or do these stimuli act on a specific network? Is the effect of painful stimulation similar to that of acupuncture? We administered the attention network test to 30 participants (15 males) to investigate the relative efficiencies of three independent attention networks (alerting, orienting, and executive control networks) under three conditions: baseline, after painful stimulation, and after acupuncture. The degree of pain experienced was assessed on a horizontally oriented visual analogue scale. The results showed that painful stimulation and acupuncture had similar effects on the orienting and executive control networks; however, there was a significantly different effect between the three conditions on the alerting network. In conclusion, (1) painful stimulation can selectively impact attention; (2) acupuncture can also selectively impact attention; i.e., both have selective influences on the alerting and executive control networks, but not on the orienting network; (3) the effects of acupuncture and painful stimulation are not identical. The mechanisms by which painful stimulation and acupuncture influence attention warrant further research.

## Introduction

### Pain

Pain is one of the most studied topics in neuroscience, and an almost ubiquitous symptom in a clinical setting. Pain has been defined as “an unpleasant sensory and emotional experience associated with actual or potential tissue damage, or described in terms of such damage” by the International Association for the Study of Pain [1]. It signals danger, and urges action to avoid further damage. When the body is hurt, for example pricked, cut, squeezed, burned, or frozen, nociceptors in the periphery respond, and the central nervous system acts to avoid or alleviate injuries. The properties of pain are related to the underlying pathological process, with its location generally indicates where the damage has occurred.

Research has demonstrated the involvement of both the peripheral and central nervous systems in pain processing. Pain involves a multifactorial, multipathway system, and each component is thought to be regulated by different brain regions. Human brain imaging studies have identified nociceptive nerve fibers passing through the nuclei of the thalamus, and projecting to higher cortical areas via two main pathways—the lateral and the medial. The lateral pain pathway travels through the ventral posterolateral nucleus and the ventral posteromedial nucleus of the thalamus, and then projects to the primary and secondary somatosensory cortices. The medial pain pathway travels through more medial cortical areas, including the anterior cingulate cortex (ACC), insula, amygdala, and hypothalamus. These two pathways are highly integrated, and both are indispensable for normal pain perception. Moreover, these areas are thought to be involved in sensory, motor, emotional, memory, and attentional processes.

### Pain and attention

It is widely known that pain serves as a warning mechanism of danger to the individual: it interrupts, distracts, and demands attention. Moreover, attention deficit is one of the most frequently reported accompanying symptoms in people experiencing either acute or chronic pain.

Many clinical studies have found that pain impacts attentional processes [2], and the brain regions where attention is modulated by pain have been identified by functional magnetic resonance imaging [3]; the ACC and thalamus are known to play key roles in both pain perception and attentional tasks, and pain and attention can both activate the same specific brain areas [4]. However, it remains to be determined whether people experiencing pain have deficits in global attention, or in a specific attention network.

### Attention networks

On the basis of many neuroanatomical and neuropsychological studies, Posner divided the human attention system into three independent networks: the alerting, orienting, and executive control networks. These networks have been distinguished at both the biochemical and cognitive levels, and have distinct neuroanatomical correlates [5-8]. The alerting network is involved in the individual’s ability to tonically maintain an alert state, and in producing a phasic response to a warning signal. Its function is critical for optimal performance. It is localized to the thalamus and frontal and parietal areas of the right hemisphere, and primarily utilizes the norepinephrine system. The orienting network is associated with selectively focusing on one, or a few items, out of many candidates. Previous studies have revealed that the orienting network consists of the temporal–parietal junction, superior and inferior parietal lobe, frontal eye fields, and the pulvinar and reticular nuclei of the thalamus, and is modulated by the cholinergic system. Finally, the executive control network refers to the ability to monitor and resolve conflicts in the presence of competing information. Neuroimaging and neuropathological studies have revealed that the ACC and lateral prefrontal cortex are involved in executive control [9-16].

The attention network test (ANT) can simultaneously measure the activity of all three networks, and evaluate their interrelationships [17], and all test results can be obtained within 20 min. Therefore, the ANT has been widely used to investigate attentional function both in healthy subjects, and in patients with disorders such as schizophrenia, Alzheimer’s disease, and borderline personality disorder, among others [18-25].

### Acupuncture and attention

Acupuncture is an ancient eastern healing methodology, which is effective in the treatment of various brain disorders, such as psychiatric diseases [26,27], insomnia [28], addiction [29,30], and stroke [31,32]. A review of the literature suggests that acupuncture can indeed influence brain activity [33-35], and modulation of several cortical and subcortical regions, including the insula, amygdala, ACC, and thalamus has been reported [36]. These regions are also highly involved in the limbic system, which plays a significant role in regulating and integrating cognition, emotion, sensory perception, and behavior. However, the neural mechanisms activated by acupuncture are extremely complicated and incompletely understood; in general, acupuncture modulates endogenous regulatory systems, including the autonomic nervous system, the endocrine system, and the neuroendocrine system, and exerts its effects not only through local reflexes but also through the central nervous system [37].

The impact of acupuncture on attention, and its three independent networks, is also unclear. Acupuncture is sometimes accompanied by the sensation of pain; however, compared to painful stimulation, acupuncture appears to have a more complex neural mechanism. Is the effect of painful stimulation similar to that of acupuncture? Do painful stimulation and acupuncture modulate all of the three attention networks, or only one or two specific networks? The present study aimed to investigate and compare the impact of painful stimulation and acupuncture on attention using the ANT. We hypothesize that: (1) painful stimulation can impact attention; (2) acupuncture can impact attention; (3) the effects of acupuncture and painful stimulation on attention are similar.

## Methods

### Subjects

In order to reduce intersubject variability, participants consisted of a homogeneous group of 30 college students [15 male, 15 females, aged 23.5 ± 1.2 (mean ± SD) years]. In order to exclude any effects of practice, we recruited another 30 college students [15 male, 15 female, aged 22.3 ± 1.4 (mean ± SD) years] as a control group to perform the ANT three times in a similar environment and on a similar schedule. All subjects were recruited from Anhui Medical University located in Hefei City, Anhui Province, China. All were right handed, and acupuncture naive. They all had normal speaking, writing, language expression, and cognitive skills. None had any history of serious physical disorders or of mental illness. Our study protocol was approved by the Anhui Medical University Ethics Committee, and written informed consent was obtained from all participants before the study.

### Procedures and measures

**Figure 1:**
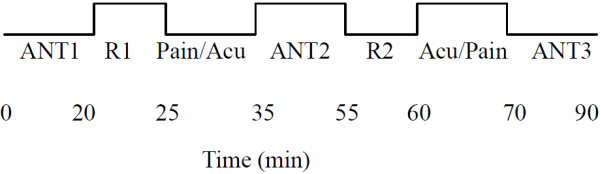
**Time course of the entire experimental procedure.** Each participant was administered the ANT three times: at baseline, after painful stimulation, and after acupuncture. First, all participants were administered the ANT1 without any interruption (Baseline). Then, all were re-administered the ANT2 immediately after painful stimulation (Pain) or after acupuncture (Acu). Lastly, all were re-administered the ANT3 immediately after acupuncture (Acu) or after painful stimulation (Pain). The sequence of Pain and Acu were counterbalanced across all participants. There was a 5-min rest period (R1, R2) after each ANT.

The experiment was divided into three blocks: baseline, after painful stimulation, and after acupuncture. During each block, all participants were administered the same ANT task. To exclude any interference of painful stimulation and acupuncture on the baseline block, each experiment began with the baseline block. Then, all participants were re-administered the ANT, immediately after either painful stimulation or after acupuncture. Lastly, the ANT was re-administered immediately after the remaining stimulus condition. In order to reduce possible influences on the results, participants were not informed of the order in which painful stimulation and acupuncture blocks would be performed, and in order to facilitate blinding, they were instructed to keep their eyes closed to prevent them from actually observing the procedures, and were asked to remain relaxed without engaging in any mental tasks. The sequence of painful stimulation and acupuncture was counterbalanced across all participants. There was a 5-min rest period after each ANT (Figure 1).

### Baseline condition

Unlike the painful stimulation and acupuncture blocks, during the baseline condition, all participants were administered the ANT task without any interruption. We designed the baseline block as the control condition.

### Painful stimulation condition

**Figure 2:**
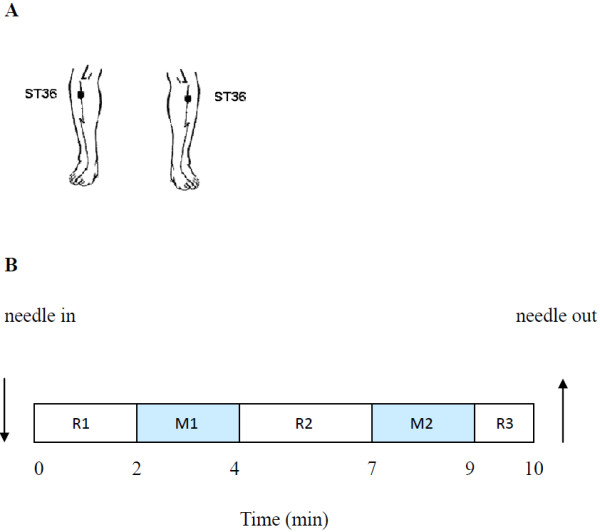
**Painful stimulation and acupuncture conditions: A ST36: 5 mm below the head of the fibula under the knee joint, and 2 mm lateral to the anterior tubercle of the tibia. B** shows the paradigm for painful stimulation and acupuncture: The needle remained at rest for 2 min (R1) before bidirectional rotation at 1 Hz for 2 min (M1). The needle was not manipulated for 3 min (R2), and then, manipulation was repeated for 2 min (M2), followed by a third period of rest for 1 min (R3). Then the needle was removed.

The acupuncture needles (sterile disposable stainless steel acupuncture needle, 0.3 mm in diameter and 40 mm in length) were inserted in 2 non-meridian points (2–3 cm away from ST36 [Stomach 36, Zusanli] near bilateral ST36 acupoints). The needles were inserted vertically, to a depth of 30 mm (Table 1). Subjects felt pain, but reported no feelings of soreness, fullness, heaviness or numbness. The needle remained at rest for 2 min (R1) before bidirectional rotation at 1 Hz for 2 min (M1). The needle was not manipulated for 3 min (R2), and then, manipulation was repeated for 2 min (M2), followed by a third period of rest for 1 min (R3), then the needle was removed (Figure 2).

**Table 1:** Procedure used for the painful stimulation and acupuncture conditions

	**Painful stimulation**	**Acupuncture**
Location	non-meridian points (2–3 cm away from ST36)	ST36
Needle gauge	0.3 mm in diameter and 40 mm in length	0.3 mm in diameter and 40 mm in length
Needle insertion depth	under the skin 30 mm	under the skin 30 mm
Insert direction	vertically	vertically

**Figure 3:**
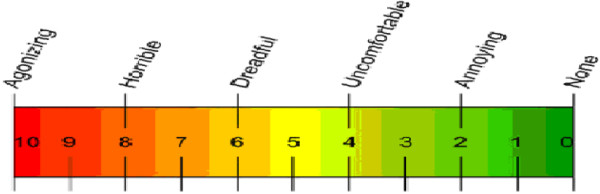
**Visual Analogue Scale of pain (VAS).** VAS is presented as a ruler with a movable cursor, the length being approximately 10 cm. “0” represents “No pain,” while “10” represents “Agonizing.” When the test began, subjects moved the cursor to the position on the ruler that best represented the degree of pain they were currently experiencing.

The entire procedure took 10 min. We used the horizontally oriented visual analogue scale (VAS) to assess the degree of pain experienced [38,39], with the mean VAS score being 5.23 ± 0.49 (mean ± SD) (Figure 3).

### Acupuncture condition

The acupuncture needles (sterile disposable stainless steel acupuncture needle, 0.3 mm in diameter and 40 mm in length) were inserted in 2 ST36 acupoints (5 mm below the head of the fibula under the knee joint, and 2 mm lateral to the anterior tubercle of the tibia) (see Figure 2A). The needles were inserted vertically, to a depth of 30 mm. The subjects felt “De-qi” sensations, such as soreness, fullness, heaviness, and numbness. The manipulation and procedure were identical to that applied in the painful stimulation condition (see Figure 2B). To exclude the feeling of dull or sharp pain, we again used the VAS to assess the degree of pain experienced during acupuncture, with the mean VAS score being 1.24 ± 0.27 (mean ± SD). This was significantly different from the painful stimulation condition (T = 35.877, *P* < 0.001). The procedure was performed by the same experienced and licensed acupuncturist on all subjects.

### Control group

In order to exclude the effects of practice from the experiment, we recruited another 30 college students as a control group, to perform the ANT three times in a similar environment and on a similar schedule.

### Between-subject comparison

In order to clarify whether there were any potential carryover effects between the painful stimulation and acupuncture conditions, we conducted a comparison between the 15 subjects who performed pain stimulation test first, and the 15 subjects who performed the pain stimulation test after acupuncture. We also compared the 15 subjects who performed the acupuncture test first, and the 15 subjects who performed the acupuncture test after pain stimulation.

### Attention network test

**Figure 4:**
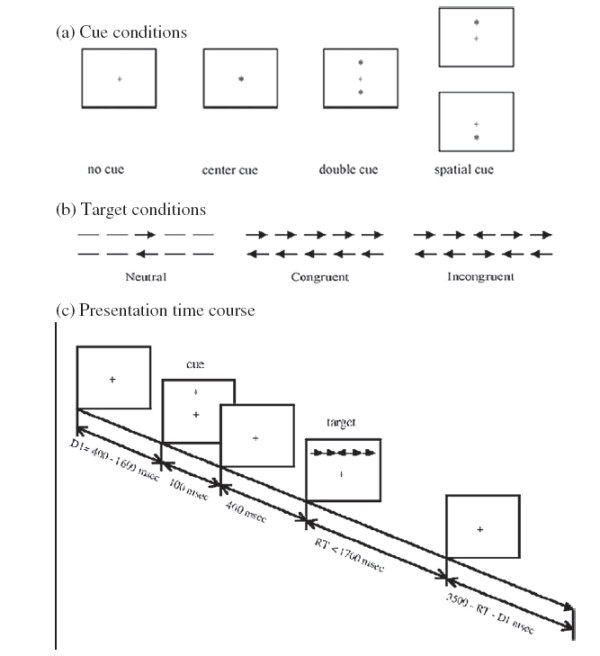
**Experimental paradigm for the attention network test.** (**a**) The four cue conditions. (**b**) The six stimuli used in the present experiment. (**c**) An example of the procedure and the time course of a trial using a spatial cue with incongruent flankers.

The attention network test was created using E-Prime (Version 1.1, Psychology Software Tools, Pittsburgh, PA, USA). Participants viewed the stimuli shown on a computer screen, and responses were automatically collected via two response buttons. The stimuli consisted of a row of five horizontal black lines, with arrowheads pointing left or right, and the target was a left or right pointing arrowhead in the center, against a gray background. The target stimulus was flanked on either side by two arrows pointing either in the same direction (congruent condition), or in the opposite direction (incongruent condition), or by nothing (neutral condition). Participants were instructed to focus on a centrally located stationary cross throughout the task, and to respond as quickly and accurately as possible. The participant’s task was to identify the direction of the center arrow by pressing one button for the left direction with the index finger of their left hand, or a second button for the right direction with the index finger of their right hand. The target stimulus remained on the screen until the participant responded, but the maximum response time was cut off at 1700 ms. Cues consisted of an asterisk appearing for 100 ms, presented 400 ms before the presentation of the target. There were four cue conditions in the process: (1) no cue, the participant was shown a cross at the same location as the first stationary cross for 100 ms; (2) a center cue, an asterisk was presented on the central point; (3) a double cue, an asterisk was presented at two target locations simultaneously, above and below the central point; and (4) a spatial cue, an asterisk was presented at a target location either above or below the central point. The whole process of the ANT consisted of a 24-trial practice block, and three experimental blocks of trials. The presentation of trials was in a random order for each participant. Each experimental block consisted of 96 trials (48 different conditions: 4 cue types × 2 target locations × 2 target directions × 3 congruencies, with 2 repetitions). The entire ANT was completed in 20 min (Figure 4).

### Calculation of attention network efficiencies

The ANT makes use of differences in reaction times (RTs) derived from the different experimental conditions to measure the efficiency of the alerting, orienting, and executive control networks [17]. Alerting efficiency was calculated by subtracting the mean RTs of the conditions with double cues from those of the conditions with no cue, as neither of these conditions provided any information on the spatial location of the target. Similarly, orienting efficiency was calculated by subtracting the mean RTs of the conditions with spatial cues from those of the conditions with center cues. In both conditions, the subject was alert, but only the spatial cue provided orientation information. Likewise, executive control efficiency was calculated by subtracting the mean RTs of congruent target conditions from those of incongruent target conditions.

### Data analysis

Statistical analysis was performed using SPSS (Version 13.0, Statistical Program for Social Sciences, SPSS Inc., Chicago, IL, USA). The statistical significance of differences between the baseline, after painful stimulation, and after acupuncture conditions was evaluated by a one-way analysis of variance (ANOVA); the between-subject comparison was evaluated using an independent samples t-test, and the threshold for statistical significance was set at *P* < 0.05. To assess differences between individual conditions, a Student–Newman–Keuls (SNK) test was also used.

## Results

### Reaction times and accuracy

The mean RTs and accuracy rates for each of the three conditions are summarized in Table 2. During the experiment, any incorrect or missed responses were excluded from the data set. We conducted a 4 (cue condition: center cue, double cue, no cue, spatial cue) × 3 (flanker type: congruent, incongruent, neutral) repeated measures ANOVA on the RT data listed in Table 2. There was a significant main effect for both cue condition and flanker type ([i] baseline: cue condition, F (3, 87) = 147.225, *P* < 0.001; flanker type, F (2, 58) = 345.498, *P* < 0.001; interaction of cue conditions × flanker types, F(6, 174) = 8.626, *P* < 0.001; [ii] after painful stimulation: cue condition, F (3, 87) = 167.751, *P* < 0.001; flanker type, F (2, 58) = 382.327, *P* < 0.001; interaction of cue condition × flanker type, F(6, 174) = 18.639, *P* < 0.001; and [iii] after acupuncture: cue condition, F (3, 87) = 250.158, *P* < 0.001; flanker type, F (2, 58) = 415.649, *P* < 0.001; interaction of cue condition × flanker type, F(6, 174) = 20.530, *P* < 0.001).

**Table 2:** Mean reaction times and accuracy under each cue condition for baseline, after painful stimulation, and after acupuncture

**Condition**	**No cue**			**Double cue**			**Center cue**			**Spatial cue**		
	**Congruent**	**Incongruent**	**Neutral**	**Congruent**	**Incongruent**	**Neutral**	**Congruent**	**Incongruent**	**Neutral**	**Congruent**	**Incongruent**	**Neutral**
Mean RTs (ms) and standard deviations												
Baseline	609 (82)	715 (93)	563 (69)	570 (74)	697 (87)	521 (66)	575 (77)	706 (90)	531 (69)	543 (72)	640 (86)	493 (59)
After painful stimulation	593 (75)	686 (87)	557 (61)	554 (69)	664 (86)	497 (63)	5560 (75)	673 (85)	500 (64)	524 (74)	608 (93)	476 (62)
After acupuncture	592 (82)	686 (84)	553 (72)	545 (76)	656 (89)	486 (64)	547 (76)	664 (91)	493 (71)	516 (77)	597 (91)	469 (62)
ACC and standard deviations												
Baseline	0.99 (0.01)	0.97 (0.04)	0.99 (0.01)	0.99 (0.01)	0.98 (0.03)	1.00(0.00)	0.99 (0.01)	0.97 (0.05)	0.99 (0.02)	1.00 (0.00)	0.98 (0.03)	0.99 (0.01)
After painful stimulation	0.99 (0.01)	0.97 (0.05)	0.99 (0.01)	0.99 (0.01)	0.98 (0.03)	1.00 (0.00)	1.00 (0.00)	0.97 (0.06)	0.99 (0.01)	0.99 (0.01)	0.99 (0.02)	0.99 (0.01)
After acupuncture	0.99 (0.01)	0.96 (0.06)	0.99 (0.01)	0.99 (0.01)	0.98 (0.03)	0.99 (0.00)	1.00 (0.00)	0.96 (0.07)	0.99 (0.01)	0.99 (0.01)	0.99 (0.02)	1.00 (0.00)

We conducted an analysis of variance with repeated measures for accuracy as well, but there were no significant main effects of cue conditions or flanker type in the three conditions (Table 2).

### Effects of painful stimulation and acupuncture on the three attention networks

**Figure 5:**
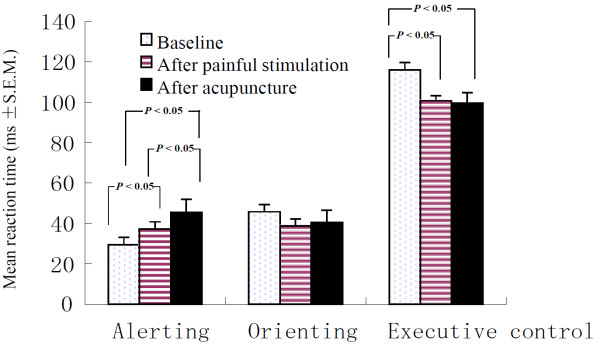
**Selective modulation of the three attention networks after the painful stimulation and acupuncture conditions.** Bar chart shows means and standard errors. The results show that with regards to alerting, participants were significantly more vigilant after either the painful stimulation or acupuncture conditions than in the baseline condition (SNK, *P* < 0.05), and the comparison between the painful stimulation and acupuncture conditions also showed a significant difference (SNK, *P* < 0.05). With regards to executive control, participants had less difficulties in resolving conflict after either painful stimulation or acupuncture than under baseline conditions (SNK, *P* < 0.05), and there was no significant difference between the painful stimulation and acupuncture conditions (SNK, *P* > 0.05). With regards to orienting, there was no significant difference in performance between any of the three conditions (SNK, *P* > 0.05).

The effects of the three attention networks in three conditions are summarized in Table 3. There was a significant main effect of the three conditions on the alerting network tasks (F (2, 87) = 9.200, *P* < 0.001). Our results show that participants were significantly more vigilant following painful stimulation or acupuncture than in the baseline condition (SNK, *P* < 0.05), and the comparison between painful stimulation and acupuncture conditions revealed a significant difference (SNK, *P* < 0.05). There was a significant main effect of the three conditions on the executive control network tasks (F(2, 87) = 8.811, *P* < 0.01). Participants had less difficulties in resolving conflict after either painful stimulation or acupuncture than in the baseline condition (SNK, *P* < 0.05), while there was no significant difference between the painful stimulation and acupuncture conditions (SNK, *P* > 0.05). However, there were no significant differences between the three conditions on the orienting network tasks (F(2, 87) = 2.398, *P* > 0.05). Thus, participants exhibit specific improvements in the performance of the alerting and executive control networks after either painful stimulation or acupuncture, but there was no impact on the function of the orienting network. There also was a significant main effect of the three conditions on the overall mean RTs (F(2, 87) = 23.238, *P* < 0.01). Participants took significantly less time to finish the test following either painful stimulation or acupuncture than they did in the baseline condition (SNK, *P* < 0.05). There was no significant difference between overall mean RTs after either of the two interferences (SNK, *P* > 0.05). Additionally, there was no significant difference observed in response accuracy between the three conditions (F (2, 87) = 0.811, *P* > 0.05) (Figure 5).

**Table 3:** Attention network scores (in RT and ratio score) of baseline, after painful stimulation, and after acupuncture

	**Baseline**		**After painful stimulation**		**After acupuncture**	
	**Mean**	**SE**	**Mean**	**SE**	**Mean**	**SE**
Alerting (ms) RT	29.43	3.73	37.27	3.66	45.47	3.54
Ratio	0.05	0.01	0.07	0.01	0.08	0.01
Orienting (ms) RT	45.8	3.59	38.83	3.34	40.6	2.46
Ratio	0.08	0.01	0.07	0.01	0.07	0.00
Executive control (ms) RT	116.17	6.57	100.8	5.93	99.73	5.02
Ratio	0.2	0.01	0.18	0.01	0.18	0.01
Accuracy (%)	98.63	0.2	98.8	0.24	98.6	0.26
Overall mean (ms) RT	594.8	13.36	572.37	13.16	565.1	13.58

### Correlation between the VAS and the attention networks

We examined the relationship between the VAS and the scores of the three attentional network tests. No significant correlations were identified between the VAS and the three network scores for either the painful stimulation or the acupuncture condition ([i] the painful stimulation: VAS and alerting, *r* = 0.218, *P* > 0.05; VAS and orienting, *r* = 0.097, *P* > 0.05; VAS and executive control, *r* = 0.273, *P* > 0.05; [ii] the acupuncture condition: VAS and alerting, *r* = 0.065, *P* > 0.05; VAS and orienting, *r* = 0.071, *P* > 0.05; VAS and executive control, *r* = −0.125, *P* > 0.05).

### Control group results

There were no significant differences observed in alerting, orienting, and executive control networks between the three trials performed by the control group ([i] Alerting: F (2, 87) = 0.070, *P* > 0.05; [ii] Orienting: F (2, 87) = 0.361, *P* > 0.05; [iii] Executive control: F (2, 87) = 0.168, *P* > 0.05).

### Between-subject comparison results

There were no significant differences observed in the alerting, orienting, or executive control networks in a between-subject comparison of the pain stimulation and the acupuncture conditions ([i] pain stimulation: Alerting, T = 0.364, *P* > 0.05; Orienting, T = 0.348, *P* > 0.05; Executive control, T = 0.071, *P* > 0.05); [ii] acupuncture condition: Alerting, T = 0.006, *P* > 0.05; Orienting, T = 0.921, *P* > 0.05; Executive control, T = 0.795, *P* > 0.05).

## Discussion

Our study used the ANT to measure participant’s performance under baseline conditions, after painful stimulation, and after acupuncture, in order to investigate how painful stimulation and acupuncture might impact the three distinct networks of attention. Painful stimulation was used as an independent variable, and the subjective experience of pain as measured by VAS was significantly different between the painful stimulation and acupuncture conditions. No significant correlations were identified between VAS and the three network scores for either the painful stimulation or the acupuncture conditions. Our experimental results exclude any significant effects of practice on the outcome, and confirmed there were no carryover effects between the painful stimulation and acupuncture conditions. We found that there were significant effects of both pain and acupuncture stimuli on the alerting network, as well as on the executive control network. In contrast, no effect of either was found on the orienting network. Moreover, a participant’s performance demonstrated that acupuncture increased alertness to a greater extent than did painful stimulation. These results suggest that painful stimulation and acupuncture both exert effects on the alerting and executive control attention networks, but not on the orienting network; they also indicate that, in the alerting network, the effect of acupuncture is quantitatively different from that of painful stimulation.

Several previous studies have shown that pain influences cognitive abilities [40-42]. As expected, the present study showed that alerting functions increased after the administration of either painful stimulation or acupuncture, with participants becoming more vigilant than they were under baseline condition. These stimuli may place them in a vigilant state, thereby ensuring that they are capable of reacting to any warning signal that may require an immediate response. Some patients with chronic pain have indeed exhibited an attentional bias, or hypervigilance for painful stimuli, which is believed to play a role in the development and maintenance of chronic pain states [43,44]. Several authors have found that an association exists between pain and hypervigilance [45,46]. One such study found that subjects showed an increased level of attention in a semantic task, which consisted of either word generation (category fluency) or word repetition, while they were receiving a painful stimulus [47].

Neural activity in several brain regions is increased in painful conditions as compared to pain-free conditions, and many imaging studies have demonstrated a minimal involvement of the prefrontal cortex, and particularly the orbitofrontal cortex and thalamus [48]. As we know, the alerting network is localized to the thalamus, frontal and parietal areas of the right hemisphere, involves the cortical projections of the norepinephrine system [49], and is responsible for activating and maintaining a vigilant state. When faced with pain, the body comes into a state of emergency, with an increase in norepinephrine secretion, which might lead to excessive norepinephrine levels in the prefrontal cortex. This might induce hypervigilance after pain. Alternatively, one study suggested that people have a limited capacity for attention, with pain being an extremely powerful noxious stimulus that demands attention, decreasing the resources available to perform other cognitive functions [50].

In our study, the performance of the executive control network was significantly decreased after both the painful stimulation and acupuncture conditions. Consistent with our first and second hypotheses, our results indicate that participants resolve conflicts more rapidly after either painful stimulation and acupuncture than they do under baseline conditions. As part of attentional processes, the executive control network is responsible for monitoring and resolving conflicts. On the basis of neuroanatomical and neurotransmitter research, the executive control network has been localized to the midline frontal areas, such as the ACC and prefrontal cortex, is modulated by dopamine (DA), and acts to monitor and resolve conflicts between competing information. Previous studies on animals and humans have shown that the medial frontal cortex, and particularly the ACC, can greatly reinforce acute nociceptive responses; for example, formalin injections enhanced behavior with disgust experience [51,52]. Furthermore, patients with frontal lesions or cingulate resections often cannot feel pain [53]. Electroencephalographic and neuroimaging studies have revealed that there are specific nociceptive neurons in the ACC that respond to noxious stimuli [54-59]. Additionally, lesions to dopaminergic neurons [60] located in the prefrontal cortex [61] result in the impairment of the executive control network. One study suggested that an age-related improvement in the performance of executive control tasks is paralleled by changing expression levels of DA and its receptor or gene [62].

In our study, the orienting network was not affected by either painful stimulation or acupuncture. This is in agreement with the findings of several other studies. For example, no difference was found in the function of the orienting network from 6 years of age to adulthood, the findings indicating that the orienting network as assessed by the ANT remains stable during brain development [21], and children with idiopathic generalized epilepsy had no deficit in the orienting network either [63]. However, some authors have suggested that exogenous orienting would be enhanced when the cue was painful [64]. In contrast to this, it has been shown that neuronal responses to nociceptive stimuli are weaker and less effective than responses to anti-nociceptive stimuli in orienting attention [65]. In our present study, the orienting network was not affected by either painful stimulation or acupuncture, but its mechanisms are not well understood, and warrant further research in the future.

In our present study, the results confirmed that acupuncture can impact selective attention networks, enhancing the efficiency of the alerting and executive control networks; notably, when compared to painful stimulation, acupuncture had a significantly greater effect on the alerting network. This is contrary to our third hypothesis; however, the mechanisms underlying this difference have yet to be elucidated. While the neural mechanisms of acupuncture are unknown, it has been suggested that it may induce an increased release of endorphins, serotonin, norepinephrine, or γ-aminobutyric acid [66]. Acupuncture might improve cognitive function by influencing neurotransmitter levels. The exact interaction between painful stimulation and acupuncture warrant further research in the future.

### Limitations

There are a number of potential limitations to the current study. We included a 5-min rest period after each ANT; it is possible that this period was not long enough for subjects to rest well. Future studies could include a longer rest period between trials.

In conclusion, the results of the current investigation support the existence of selective effects on the alerting and executive control attentional networks following either painful stimulation or acupuncture. To the best of our knowledge, this is the first study to concurrently assess the effects of both painful stimulation and acupuncture on the three attention networks, using the ANT. From a clinical/functional point of view, we confirmed that: (1) painful stimulation can impact attention; (2) acupuncture can impact attention; (3) the effects of acupuncture and painful stimulation are not identical. Further studies could combine the ANT with functional magnetic resonance imaging and positron emission computed tomography in order to more precisely investigate the neural mechanisms underlying the influence of painful stimulation and acupuncture on functional attention networks.

## Competing interest

The authors have declared that they have no competing interest.

## Authors’ contributions

Conceived and designed the experiments: GL, JF, KW. Performed the experiments: GL, HJM, SH, YHT. Analyzed the data: GL, PPH. Wrote the paper: GL. All authors read and approved the final manuscript.
